# Economic analysis of remote monitoring in patients with implantable cardioverter defibrillators or cardiac resynchronization therapy defibrillators in the Trento area, Italy

**DOI:** 10.3389/fcvm.2023.1151167

**Published:** 2023-05-25

**Authors:** Massimiliano Marini, Lodovica Videsott, Chiara Francesca Dalle Fratte, Andrea Francesconi, Eleonora Bonvicini, Silvia Quintarelli, Marta Martin, Fabrizio Guarracini, Alessio Coser, Pier Paolo Benetollo, Roberto Bonmassari, Giuseppe Boriani

**Affiliations:** ^1^ Department of Cardiology, S. Chiara Hospital, Trento, Italy; ^2^ Controlling Department, APSS (Azienda Provinciale per i Servizi Sanitari), Trento, Italy; ^3^ Department of Management and Economy, University of Trento, Trento, Italy; ^4^ Cardiology Division, Department of Biomedical, Metabolic and Neural Sciences, University of Modena and Reggio Emilia, Policlinico di Modena, Modena, Italy

**Keywords:** remote monitoring, heart failure, hf, cardiac implantable electronic device, CIED, Italy

## Abstract

**Introduction:**

Remote monitoring (RM) technologies have the potential to improve patient care by increasing compliance, providing early indications of heart failure (HF), and potentially allowing for therapy optimization to prevent HF admissions. The aim of this retrospective study was to assess the clinical and economic consequences of RM vs. standard monitoring (SM) through in-office cardiology visits, in patients carrying a cardiac implantable electronic device (CIED).

**Methods:**

Clinical and resource consumption data were extracted from the Electrophysiology Registry of the Trento Cardiology Unit, which has been systemically collecting patient information from January 2011 to February 2022. From a clinical standpoint, survival analysis was conducted, and incidence of cardiovascular (CV) related hospitalizations was measured. From an economic standpoint, direct costs of RM and SM were collected to compare the cost per treated patient over a 2-year time horizon. Propensity score matching (PSM) was used to reduce the effect of confounding biases and the unbalance of patient characteristics at baseline.

**Results:**

In the enrollment period, *N* = 402 CIED patients met the inclusion criteria and were included in the analysis (*N* = 189 patients followed through SM; *N* = 213 patients followed through RM). After PSM, comparison was limited to *N* = 191 patients in each arm. After 2-years follow-up since CIED implantation, mortality rate for any cause was 1.6% in the RM group and 19.9% in the SM group (log-rank test, *p* < 0.0001). Also, a lower proportion of patients in the RM group (25.1%) were hospitalized for CV-related reasons, compared to the SM group (51.3%; *p* < 0.0001, two-sample test for proportions). Overall, the implementation of the RM program in the Trento territory was cost-saving in both payer and hospital perspectives. The investment required to fund RM (a fee for service in the payer perspective, and staffing costs for hospitals), was more than offset by the lower rate of hospitalizations for CV-related disease. RM adoption generated savings of −€4,771 and −€6,752 per patient in 2 years, in the payer and hospital perspective, respectively.

**Conclusion:**

RM of patients carrying CIED improves short-term (2-years) morbidity and mortality risks, compared to SM and reduces direct management costs for both hospitals and healthcare services.

## Introduction

Management of acute cardiovascular diseases is constantly improving, which leads to a progressive increase of life expectancy and consequently, to a progressive increase of disease prevalence (1). Unavoidably, this will be associated with an increase of direct and indirect cost. Over a period of 20 years (2010–2030) a +25% increase of prevalence is expected to generate an increase of +215% of costs (2). Therefore, it is of paramount importance to explore innovative solutions that hold the promise of improving clinical outcomes in the domain of cardiovascular diseases and may reduce the financial burden on healthcare systems.

Heart failure (HF) is the largest cause of hospitalization in patients aged ≥65 years in Western countries (3–5). Recently data from the Italian ARNO observatory estimated that patients hospitalized for HF in Italy have 56% risk to experience a new hospital admission within one year since their discharge (6); interestingly, almost half of these new hospital admissions (49%) are due to non-cardiovascular reasons, meaning that patients with cardiovascular disease require a comprehensive, multidisciplinary monitoring. The same study showed that the Italian National Healthcare Service (NHS, or SSN -Servizio Sanitario Nazionale-) spends about €550 million every year for these patients, with each hospital admission costing €11,867, and with hospital readmissions being approximately twice more expensive than first admissions (6).

Management of HF is complex, quite often, patients who carry cardiac implantable electronic devices (CIEDs) also present other chronic conditions (such as diabetes, chronic obstructive pulmonary disease, cognitive impairment, osteo-articular diseases, etc.), have reduced life expectancy, poor quality of life, and the highest risk of hospital admission among any other disease in the western world (7).

An increasing number of patients with heart failure receive implantable cardioverter-defibrillators (ICDs) or cardiac resynchronization defibrillators (CRT-Ds) with remote monitoring (RM) function. Early detection of worsening heart failure, enabled by the regular collection and monitoring of predisposing factors or symptoms (such as weight, heart rate, and blood pressure) can help patients and physicians to adjust therapy or timely intervene in case of anomalies, thus improving clinical outcomes (8, 9). Also implanted therapeutic devices can provide, wirelessly and remotely, information on the device itself (generator and lead function), that are useful to verify the appropriate functioning (8, 9).

For all these reasons, the use of RM in patients with HF, and left ventricular systolic dysfunction, treated with implantable cardioverter-defibrillators (ICDs) or cardiac resynchronization therapy defibrillators (CRT-Ds) may improve efficiency of care, drives decision making, and has the potential of reducing the burden of hospital admissions for HF and other major cardiovascular events (10, 11).

For instance, a systematic review conducted in 2017 identified 39 relevant trials of RM, using non-implanted systems and largely based on assessments of symptoms, weight, blood pressure, heart rate and rhythm (12). The meta-analysis showed that RM was associated with a reduction in all-cause mortality of 20% and HF hospitalization of 37% (12). Finally, the adoption of RM has been proven to be an efficient way of monitoring HF patients in situations during which there is an important need of reducing or interrupting face-to-face consultations, like the recent COVID-19 pandemic (13–16).

Thanks to this compelling evidence, the Healthcare Service of Trento (Italy) has made the decision of funding RM for patients who carry CIEDs and has set up an ambulatory tariff for hospitals in charge of this service.

The aim of this observational, retrospective study, promoted by the Cardiology Unit of the Santa Chiara Hospital, was to conduct an assessment on the clinical and economic consequences of remotely monitoring patients treated with ICDs or CRT-Ds, comparing two alternatives: patients monitored through remote monitoring (hereafter, “RM”), vs. patients monitored through standard, in-office cardiology visits (hereafter, “SM”).

## Material and methods

### Data

Clinical and resource consumption data for this retrospective analysis were extracted from the Electrophysiology Registry of the Trento Cardiology Unit, which has been systemically collecting patient information from January 2011 to February 2022.

The study protocol was approved by the local ethics committee. The investigation conformed to the principles outlined in the Declaration of Helsinki. All patients gave written informed consent, and data were treated confidentially.

The study population consisted of adult patients (aged ≥18 years), who: (i) received either ICD or CRT-D therapy; (ii) were discharged alive from the hospital, after implantation; (iii) were monitored with either RM or standard in-office cardiology visits; (iv) were followed up by the Electrophysiology Unit of the Santa Chiara Hospital (i.e., data were properly tracked in the registry) for at least 2 years (study period), or died before the end of the study period. Patient observation started at the date of CIED implantation, which served as index date.

Demographic and clinical characteristics of CIED implanted patients were collected to identify potential differences between the two groups (remote vs. standard monitoring; (Table 1), then clinical and economic data of these patients were collected.

**Table 1:** Summary of data collected in the Trento registry.

Data type	Description (list of items)
Demographics	Age, gender
Clinical characteristics at baseline	Diabetes mellitus, pulmonary-arterial hypertension, chronic kidney disease, history of cardio- or cerebrovascular disease (coronary-artery disease, thromboembolism or vasculopathy, atrial fibrillation, type of heart disease, stroke, ischemic transient attack, etc.), HF-related data, CHA_2_DS_2_-VASc score, echocardiographic data
CIED implantation data	Date of CIED implant, therapeutic indication (primary vs. secondary, name of the implanted device, type of electrostimulation.
Medical therapy prescribed after CIED	Anticoagulation therapy, angiotensin-converting enzyme (ACE) inhibitors, angiotensin II receptor blockers (ARBs), angiotensin receptor neprilysin inhibitors (ARNI), beta-blockers, diuretics, antiarrhythmic agents.
Treatment assignemnt	Remote monitoring or standard monitoring
Monitoring	Date of visit, type of visit (remote^a^ or in-office), type of visit (planned or urgent), diagnostic exams prescribed after visit (if applicable).
Hospitalization	Date of hospital admission, reason of hospital admission, length of stay, ward, diagnostic exams prescribed during hospitalization (if applicable), invasive procedures (e.g., by-pass graft, percutaneous intervention, device upgrade, etc.) during hospitalization (if applicable).
Death	Date of death

CHA_2_DS_2_-VASc score, Congestive heart failure, Hypertension, Age over 75 years, Diabetes mellitus, Stroke, Vascular disease, Age between 65 and 74 years, Sex Category (female); CIED, cardiac implantable electronic devices, HF, Heart Failure; RM, remote monitoring.

^a^ By definition, remote visit was possible only the RM group, while in-office visit was possible in both groups.

The Remote Monitoring is carried out daily (weekdays) and provides for the analysis of alarms according to a color code. It is performed by two appropriately trained dedicated nurses who consult with the electrophysiologist cardiologist as needed. The basic parameters of the devices were always re-checked during the visit in presence (sensing, impedances, threshold, programming, battery life).

The physicians involved in remote monitoring activities are the same ones who also carry out outpatient visits and they are part of the electrophysiology team of the Trento Hospital Cardiology.

Patients with a defibrillator undergo a scheduled outpatient visit once a year. Extra visits (unscheduled visits) were based on the severity of the remote monitoring alarm.

From a clinical standpoint, survival analysis was conducted, and incidence of CV-related hospitalizations was measured. From an economic standpoint, direct costs (RM implementation, planned and unplanned in-office visits, laboratory and instrumental diagnostic examinations, hospital admissions) were summed up to estimate the cost per treated patient over a 2-year time horizon, and adopting two different perspectives: (i) the payer perspective (Trento Healthcare Service); (ii) the provider perspective (Santa Chiara hospital).

### Cost analysis: payer (healthcare service) perspective

Table 2a shows the unit costs that were used to estimate the economic impact adopting the payer perspective. The payer perspective captured the costs the Trento Healthcare Service covered to manage the study population during the 2-year observation period. These costs depend on the current tariffs (for both inpatient and outpatient services) the Healthcare Service remunerates hospitals and other healthcare providers with. For the inpatient care, the cost of hospital admissions corresponds to the DRG tariffs issued by the Healthcare Service *[“Nomenclatore tariffario delle prestazioni di ricovero per acuti della Provincia Autonoma di Trento”*(17)]; similarly, for outpatient care, the cost of visits, exams, etc. was retrieved from the formulary of outpatient services issued by the Healthcare Service *[“Nomenclatore tariffario delle prestazioni di assistenza specialistica ambulatoriale della Provincia Autonoma di Trento”*(18)].

Two different providers perform control visits in the Trento territory: (i) the heart failure (HF) ambulatory; (ii) the electrophysiology (EF) ambulatory. The associated cost of the two visits is slightly different, and such difference was captured in the analysis. The cost of the in-office visit in the HF ambulatory amounts to €25.70, consisting in a control visit (€12.90; Code 89.01.3), plus electrocardiogram (ECG; €12.80; Code 89.52). The cost of the in-office visit in the EF ambulatory amounts to €38.45, consisting in a control visit (€12.90; Code 89.01.3), plus pacemaker control (€25.55; Code 89.48.1) (18).

### Cost analysis: provider (hospital) perspective

The provider perspective captured the production costs the hospitals sustain to deliver care to the CIED patients. The inpatient costs were calculated by multiplying the daily cost of hospital stay (depending on the ward; as calculated by the management & control department of the Santa Chiara Hospital, Trento), by the length of each hospitalization. Table 2b shows the hospital daily costs, recently calculated by the hospital [2019 (19)]). The cost of provision of outpatient and in-office visits was calculated considering staffing costs (€73/hour for physicians and €28/hour for assistant nurses). It was assumed that the duration of an in-office visit was half an hour, and that both a physician and a nurse would be needed during the visit. Since it was not possible to accurately determine the fixed costs of ambulatory care (i.e., room occupation), it was assumed that they would have minimal impact on the total cost of healthcare provision, and they were calculated as 10% mark-up of the staffing costs. The resulting cost was €55.55 per in-office visit (€73/hour × 0.5 h + €0.28/hours × 0.5 h), in the hospital perspective. The cost of RM, amounting to €68.20 per patient/year, was calculated taking into account that, a patient population of about *N* = 900 patients would require 1 full-time equivalent nurse (∼€45.3 thousands/year for a nurse paid €28/hour for 7.36 h/day and 220 working days/year) and 1 h-time equivalent physician (∼€16.1 thousands/year for a physician paid €73/hour for 1 h/day and 220 working days/year).

**Table 2:** Unit costs in the payer/healthcare service (a) and provider/hospital (b) perspective.

(a) Payer/healthcare service perspective Item	Unit cost (€)^a^	Source (17, 18)
Intracranial hemorrhage or cerebral infarction	4,740.66	DRG 14
Acute cerebrovascular diseases	3,171.05	DRG 15
Infections and respiratory inflammation, with cc	10,712.42	DRG 79
Pulmonary edema and respiratory disease	3,523.23	DRG 87
Chronic obstructive pulmonary disease	1,767.11	DRG 88
Pneumonia or pleuritis, with cc	5,775.06	DRG 89
Other cardio-thoracic interventions	16,581.53	DRG 108
Major interventions on the cardiovascular system, with cc	14,875.13	DRG 110
Revision of the cardiac pacemaker	6,444.49	DRG 117
Replacement of the cardiac pacemaker	12,662.37	DRG 118
Other interventions on the circulatory system	19,672.70	DRG 120
Myocardial infarction (MI) with cardiac catheter	5,899.70	DRG 122
Cardiovascular disease (excl. MI) with cardiac catheter, with cc	5,493.02	DRG 124
Cardiovascular disease (excl. MI) with cardiac catheter, without cc	2,395.76	DRG 125
Cardiogenic shock	4,089.12	DRG 127
Atherosclerosis, with cc	658.50	DRG 132
Atherosclerosis, without cc	313.64	DRG 133
Cardiac congenital valvular disease, with cc	4,195.44	DRG 135
Arrhythmias, with cc	4,902.64	DRG 138
Arrhythmias, with cc	264.04	DRG 139
Angina pectoris	3,005.41	DRG 140
Syncope and collapse	3,332.28	DRG 141
Thoracic pain	5,657.53	DRG 143
Other diagnoses of the cardiovascular system, with cc	6,423.33	DRG 144
Other diagnoses of the cardiovascular system, without cc	333.36	DRG 145
Endocrine diseases, without cc	1,312.21	DRG 301
Chronic kidney disease	6,015.06	DRG 316
Implantation of cardiac defibrillator, without catheter	28,582.78	DRG 515
PTCA, without stenting, without MI	7,522.89	DRG 518
Implantation of cardiac defibrillator, with catheter, with MI, cardiac failure, or cardiogenic shock	37,002.03	DRG 535
Coronary by-pass graft without catheter	22,979.93	DRG 550
Implantation of cardiac pacemaker and automatic defibrillator	16,138.86	DRG 551
PTCA, without stenting, with major cardiovascular disease	12,101.36	DRG 555
PTCA, with bare metal stenting, without major cardiovascular disease	9,527.15	DRG 556
PTCA, with drug eluted stenting, with major cardiovascular disease	14,364.23	DRG 557
PTCA, with drug eluted stenting, without major cardiovascular disease	11,721.42	DRG 558
Respiratory disease, requiring assisted ventilation (>96 h)	14,647.13	DRG 565
Respiratory disease, requiring assisted ventilation (<96 h)	7,014.38	DRG 566
Sepsis, without assisted ventilation	5,640.47	DRG 576
Electrocardiogram	12.80	Code 89.52
Cardiology visit (control)	12.90	Code 89.01.3
Pacemaker control and set-up, including electrocardiogram	25.55	Code 89.48.1
Remote control of patients carrying CIED and loop recorder	25.55	Code 89.48.2
(b) Provider/hospital perspective Item	Daily cost (€)^a^	Source (18)
Hospital admission: internal medicine	430.62	
Hospital admission: cardiology	1,625.06	
Hospital admission: infection diseases	971.54	
Hospital admission: geriatrics	286.04	
Hospital admission: nephrology	826.59	
Hospital admission: pneumology	658.15	
Hospital admission: cardiac surgery	933.32	
Hospital admission: neurology	1,010.00	

CC, complications; CIED, cardiac implantable electronic device; MI, myocardial infarction; PTCA percutaneous transluminal coronary angioplasty.

^a^ 130% of standard tariffs.

### Statistical analysis

Descriptive analyses and simple parametric and non-parametric testing (*t*-test and chi-square test) were used to identify potential differences at baseline between the RM and SM groups, as this study did not have randomized design. Because of the lack of balance for some of these characteristics (Supplementary Table S1), propensity score matching (PSM) was used to adjust the selection bias of assigning patients to the remote monitoring group. Covariates that were input in the probit regression model determining the PSM were as follows: age, gender, type of implanted device (CRT-D, cardiac resynchronization therapy defibrillator; ICD, implantable cardioverter-defibrillator), diagnosis of stroke or transient of ischemic attack, myocardial infarction, atrial fibrillation, deep vein thrombosis, aortic stenosis, category of left ventricular ejection fraction, New York Health Association (NYHA) class.

After PSM adjustment, standard statistical tests were conducted to assess potential differences among RM and SM groups. Survival curves and event rates in the matched population were estimated using the Kaplan-Meier method, and log-rank test. Binary outcomes (e.g., proportion of patients with at least one hospitalization) were compared by using non parametric test of proportions, while continuous variables (e.g., costs) were compared by using parametric *t*-test. Finally, for costs a mixed approach of testing difference with both parametric and non-parametric tests was preferred. All statistical data were analyzed using the Stata 13 software.

## Results

### Baseline characteristics and adjustment after propensity score matching

In the enrollment period (January 2011 to February 2022), *N* = 402 patients carrying either ICD or CRT-D devices met the inclusion criteria and were included in the analysis. Among the population of *N* = 402 patients overall, *N* = 189 patients (47.0% of the cohort), mainly enrolled in early years (2011 or 2012), were followed through SM, while the remaining *N* = 213 patients (53.0% of the cohort) entered the RM program, which consisted of regular RM control plus in-office visits. Given some imbalance of baseline characteristics (overall, the SM cohort seemed more severe than the RM cohort, and some differences were statistically significant at the *p* = 0.05 level; Supplementary Table S1), PSM with common support was applied to minimize such differences and minimize effect of confounding factors on the outcomes. A probit regression model was used to identify variables predicting assignment to the RM group (Supplementary Figure S1). The PSM model was efficient, with only 3 out of 194 valid RM observations being not suitable for control matching. After matching, baseline characteristics of the two groups were balanced, except for the proportion of patients treated with diuretics (Table 3).

**Table 3:** Patients’ charactristics at baseline (after propensity score macthing).

Parameter	SM group (*N* = 191)	RM group (*N* = 191)	Total (*N* = 382)	Difference (test)^a^
Age, years, mean (SD)	65.13 (12.85)	64.77 (13.16)	64.95 (12.99)	*p* = 0.7862
Male gender, %	82.72% (158/191)	78.01% (149/191)	80.37% (307/382)	*p* = 0.246
Diabetes, %	18.85% (36/191)	21.99% (42/191)	20.42% (78/382)	*p* = 0.446
Pulmonary arterial hypertension, %	59.16% (113/191)	58.64% (112/191)	58.90% (225/382)	*p* = 0.917
Severe chronic kidney disease, %	2.62% (5/191)	3.66% (7/191)	3.14% (12/382)	*p* = 0.557
Secondary cardiovascular prevention, %	39.79% (76/191)	34.55% (66/191)	37.17% (142/382)	*p* = 0.290
Stroke or TIA, %	8.38% (16/191)	7.85% (15/191)	8.12% (31/382)	*p* = 0.851
Myocardial infarction, %	31.94% (61/191)	33.51% (64/191)	32.72% (125/382)	*p* = 0.744
Coronary disease, without MI, %	16.75% (32/191)	12.57% (24/191)	14.66% (56/382)	*p* = 0.247
Thromboembolism or vasculopathy, %	3.14% (6/191)	5.76% (11/191)	4.45% (17/382)	*p* = 0.215
Deep vein thrombosis, %	65.45% (125/191)	70.16% (134/191)	67.80% (259/382)	*p* = 0.324
Aortic stenosis, %	(3/191)	2.09% (4/191)	1.83% (7/382)	*p* = 0.703
Atrial fibrillation, %	30.37% (58/191)	26.70% (51/191)	28.53% (109/382)	*p* = 0.428
Treatment with ACE inhibitors/ARBs, %	83.60% (158/189)	80.42% (152/189)	82.01% (310/378)	*p* = 0.422
Treatment with beta-blockers, %	94.18% (178/189)	94.15% (177/188)	94.16% (355/377)	*p* = 0.990
Treatment with diuretics, %	86.24% (163/189)	78.31% (148/189)	82.28% (311/378)	***p* = 0.043**
Treatment with anticoagulation drugs, %	30.89% (59/191)	37.57% (71/189)	34.21% (130/180)	*p* = 0.170
Treatment with antiarrhythmic drugs, %	16.67% (30/180)	20.21% (38/188)	18.48% (68/368)	*p* = 0.381
Treatment with ICD, %	62.83% (120/191)	63.87% (122/191)	63.35% (242/382)	*p* = 0.832
Treatment with CRT-D, %	37.17% (71/191)	36.13% (69/191)	36.65% (140/382)	
CHA_2_DS_2_-VASc score, mean (SD)	2.76 (1.66)	2.87 (1.65)	2.82 (1.66)	*p* = 0.5171
Left ventricular ejection score, %				
<35%	12.57% (24/191)	16.23% (31/191)	14.40% (55/382)	*p* = 0.172
35%–44%	14.14% (27/191)	7.85% (15/191)	10.99% (42/382)	
45%–54%	13.61% (26/191)	11.52% (22/191)	12.57% (48/382)	
>55%;	59.69% (114/191)	64.40% (123/191)	62.04% (237/382)	
NYHA Functional Classification, %				
Class I	18.85% (36/191)	19.9% (38/191)	19.37% (73/382)	*p* = 0.400
Class II	74.35% (142/191)	69.63% (133/191)	71.99% (275/382)	
Class III	6.81% (13/191)	10.47% (20/191)	8.64% (33/382)	
Class IV	0.00% (0/191)	0.00% (07191)	0.00% (0/382)	

ACE, angiotensin-converting enzyme; ARB, angiotensin II receptor blocker; CHA_2_DS_2_-VASc score, Congestive heart failure, Hypertension, Age over 75 years, Diabetes mellitus, Stroke, Vascular disease, Age between 65 and 74 years, Sex Category (female); CRT-D, cardiac resynchronization therapy defibrillators; ICD, implantable cardioverter-defibrillators; NYHA, New York Heart Association; RM, remote monitoring; SD, standard deviation; SM, standard monitoring; TIA, transient ischemic attack.

^a^ *t*-test for age and CHADS score; chi-square test for all other variables. Statistically significant differences between groups are marked in bold.

There were *N* = 42 episodes of device malfunctioning in *N* = 36 patients, of whom *N* = 13 were managed through remote control (only one third of the patients with malfunction). This suggests that RM may provide early indications of functional problems and avoid device malfunctioning. None of these *N* = 42 episodes were correlated with patient death.

There were *N* = 97 interventions of device change in *N* = 97 patients, of whom *N* = 49 were managed through remote control. The information about explant is not available in the database.

### Survival and CV-related hospitalization rates

**Figure 1:**
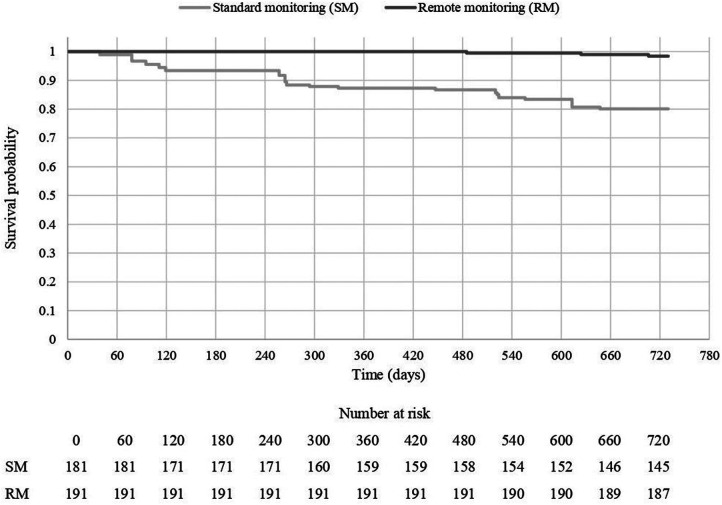
2-year mortality (any cause), by treatment group.

From a clinical perspective, the association between RM and mortality (for any cause), as well as CV-related morbidity was assessed. After a follow-up of 2 years since CIED implantation, mortality rate for any cause was 1.6% (*N* = 3 out of 191 patients) in the RM group and 19.9% (*N* = 36 out of 191 patients) in the SM group (log-rank test, *p* < 0.0001; Figure 1).

**Figure 2:**
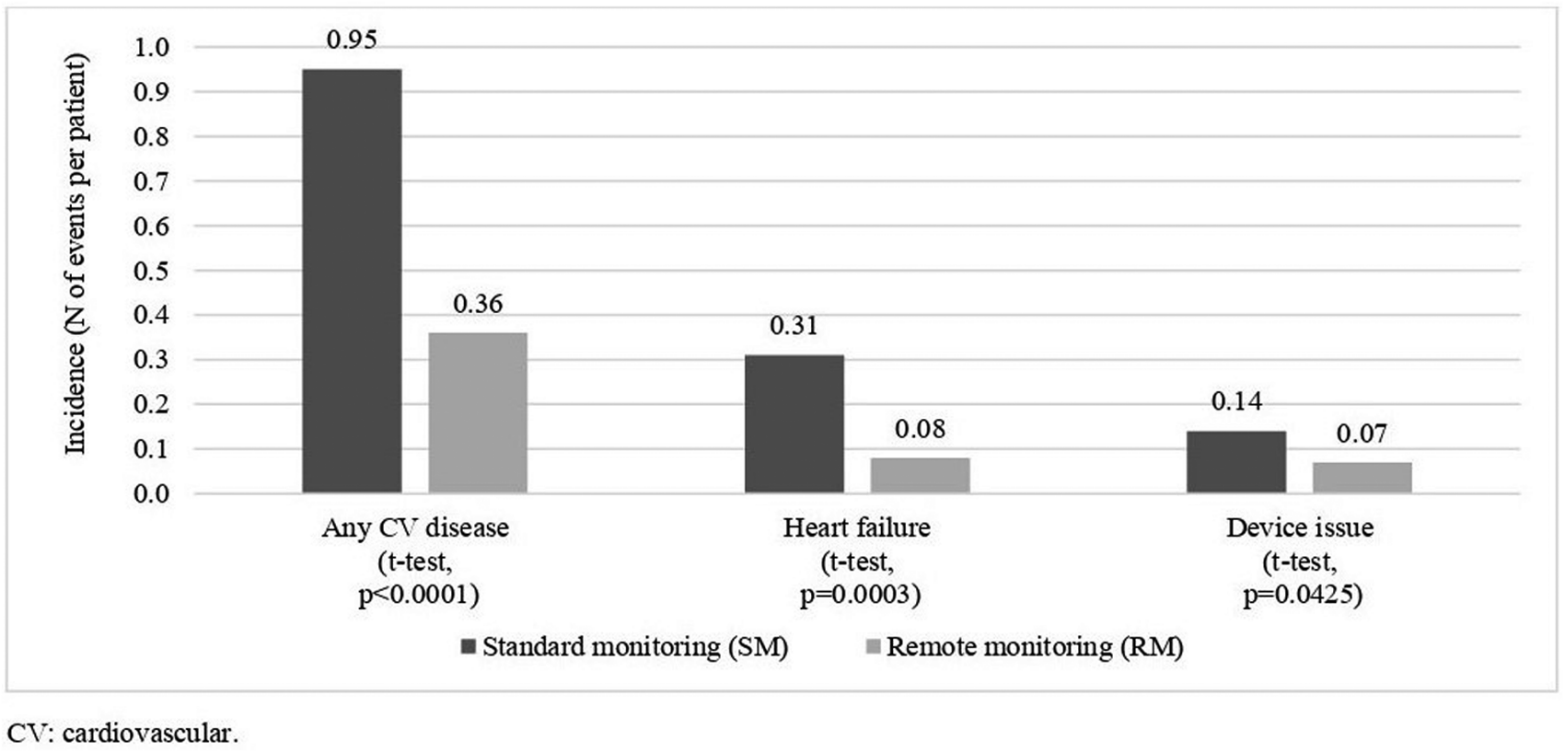
2-year CV-hospitalization incidence rates, by treatment.

During the study period, a lower proportion of patients in the RM group, 25.1% (*N* = 48 out of 191 patients) were hospitalized for CV-related reasons, compared to the SM group (51.3%; *N* = 98 out of 191 patients; *p* < 0.0001, two-sample test for proportions). A similar lower rate of hospitalizations was observed for other types of hospitalizations, such as hospitalizations for heart failure (7.3% in the RM group, 18.8% in the SM group; *p* = 0.0008) and hospitalizations for device issues (5.2% in the RM group, 13.6% in the SM group; *p* = 0.0051). Figure 2 shows the 2-year incidence rates of hospitalizations for any CV cause, for heart failure, and for device issues in the two groups. The incidence rates of CV-related and HF-related hospitalizations were significantly lower in the RM group, compared to the SM group (*p* < 0.05). The length of stay per hospital admission was similar between the two groups (6.0 days/admission in the SM group, vs. 6.8 days days/admission in the RM group (*p* = 0.3619, two-sample *t*-test).

Interestingly, a similar number of in-office visits was registered in the RM group (4.51 visits/patient the 2-year period), vs. the SM group (4.32 visits/patient; (*p* = 0.5490, two-sample *t*-test), in contrast with the expected occurrence of more visits in the RM group, triggered by device alerts, inducing physicians to request a follow-up in-office visit to examine the patient.

### Costs for inpatient care

Adopting the payer perspective, RM was associated with a lower average cost of hospital admission for any CV reason of 62% vs. SM. Over the 2-year period, costs were €2,345/patient and €7,176/patient in the RM and SM groups, respectively (difference: -€4,831; *t*-test, *p* < 0.0001). An even larger difference was also observed in the hospital perspective (where production costs, instead of DRG tariffs, were used to estimate the costs for the hospital). In the hospital perspective analysis, costs were €2,518/patient and €9,523/patient in the RM and SM groups, respectively (difference: −€-7,005; *t*-test, *p* < 0.0001). The large cost difference between the two groups was driven by the significant difference in the hospitalization rates, which favored the RM group.

### Costs for outpatient care

When tariffs were used to calculate the economic impact of outpatient care under the payer perspective, costs amounted to €147 in the SM group, and €207 in the RM group (difference: +€60; *t*-test, *p* < 0.0001), over the 2-year study period. As expected, implementation of RM lead to higher costs (+41%), that were attributable to the cost of RM visits, and to an increase of in-office visits triggered by RM control. A cost increase was also seen in terms of production costs, with costs in the RM group (€505) being 100% higher than in the SM group, €253 (difference: +€253; *t*-test, *p* < 0.0001).

### Total cost of care (inpatient + outpatient care)

**Figure 3:**
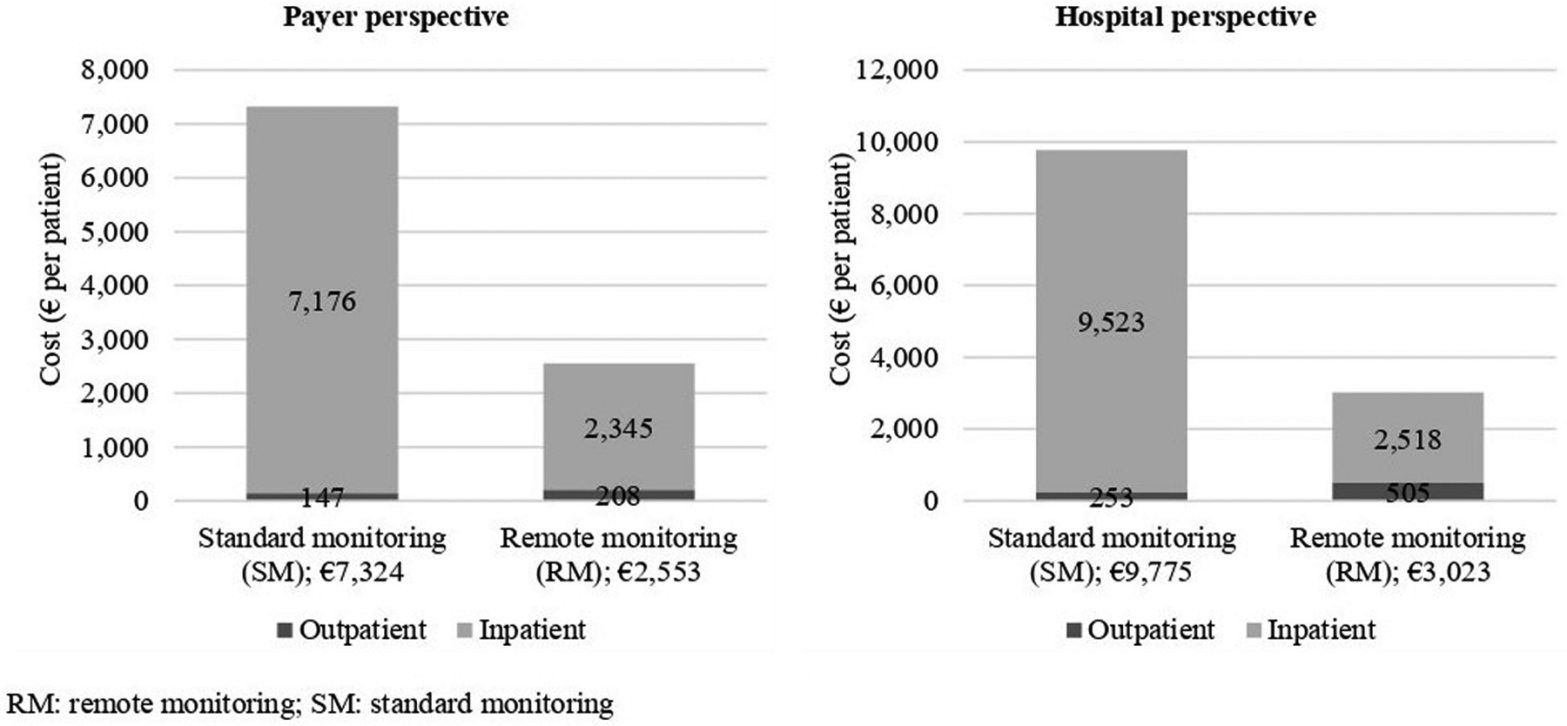
Total cost of care, by (**A**) payer and (**B**) hospital perspective.

Overall, the implementation of the RM program in the Trento territory emerged as cost saving in both payer and hospital perspectives. The relatively small investment required to fund RM (a fee for service in the payer perspective, and staffing costs for hospitals), was more than offset by the lower rate of hospitalizations for CV-related disease. Figure 3 shows that RM adoption generated savings of −€4,771 and −€6,752 per patient in 2 years, in the payer and hospital perspective, respectively.

For completeness, few non-parametric tests were conducted to confirm the cost difference between RM and SM. First, we tested the proportion of patients exceeding a certain cost threshold of €1,000 in the follow-up period; this analysis confirmed that fewer patients had a cost above this threshold in the RM group (26.7%), compared to the SM group (51.3%; *p* < 0.0001, two-sample test for proportions). Second, when patients were stratified by cost quartiles, the distribution of patients was different in the two groups, with much fewer SM than RM patients in the highest quartile (16.75% vs. 30.37%, respectively; *p* < 0.0001, Pearson chi square test).

## Discussion

The organizational model for RM management, including the identification of roles and responsibilities, the involvement of healthcare professionals, and the setup of an action plan in response to CIED system of alarms, is crucial to optimize the efficiency of this intervention, and make sure it guarantees the “promised” results, namely improvement of clinical outcomes and reduction of acute care costs. Such organizational aspects have to be carefully considered, as important elements for explaining the clinical and economic results associated with RM implementation (20).

The RM management model currently used in the Trento Cardiology Unit of the Santa Chiara Hospital is the result of progressive changes that have fixed the initial issues and inefficiencies, while integrating the constant technological improvements in the cardiology space. Scientific literature has shown that adopting an efficient model is as important as selecting the best healthcare technologies and solutions for patients; even the most effective therapeutic intervention is destined to fail if it is not used properly (21–24).

This retrospective analysis of observational data provides evidence that RM of patients carrying CIEDs can have significantly lower short-term (2-year) morbidity and mortality risks, compared to standard monitoring based on the traditional in-office visit approach. These results are in line with the IN-TIME trial (11), and highlight a benefit not demonstrated by other prospective studies on remote monitoring with implanted devices (25–27).

The immediate, plausible consequence of this lower hospitalization rate is a significant drop of the inpatient costs, which alleviates the economic burden for the healthcare service. The reduction of hospital admissions in our Trento territory has generated savings from both payer and hospital perspective. The different saving observed in the two perspectives depends on the fact that while remuneration of inpatient services, through the DRG system, is fixed within a certain length of stay, production costs for the hospital increase almost linearly with length of stay. For instance, the remuneration for an episode of heart failure (DRG 127) amounts to €4,089, regardless the length of stay (provided that this is comprised between 2 and 21 days, otherwise beyond the 21st day, an additional remuneration of €263.02 applies), while the hospital cost of a 21-day stay would be about ten times higher than that of a 2-day stay. The implementation of the RM system led to a slight increase of both payer and hospital costs. More specifically, RM increased costs by €88 per patient/year in the payer perspective, and by €100 per patient/year in the hospital perspective. The increase of visits in the RM group (+33% compared to the SM group) deviates from what observed in literature, for example, in the RM-ALONE (10) and TARIFF (28) studies. However, the data used for the present analysis refer to about 40% of the total CIED population of the Trento territory, therefore we do not know (i.e., do not have complete data) whether this trend applied to the full cohort of patients. In any case, an increase of the in-office visits is still plausible. Patients under RM are followed through a system of “alerts & alarms”, and they could be called back by their physicians because of this exchange of information, which is critical to prevent further disease worsening. However, even if the RM model could be made more efficient and less expensive (perhaps by replacing some in-office visits with phone or video calls), the investment in RM is minimal and the return of investment for avoided hospitalization clearly offsets the investment. Alternatives to conventional methods of payment and reimbursement, such as risk-sharing arrangements have been proposed (29) and could be considered also for remote monitoring, in the settings where its value is of proven benefit. As for most observational studies, certain methodological issues could limit the validity of results of the present study. First, as already mentioned, the analysis, refers to less, than half of the CIED patients receiving care in our territory. Despite the sample was relatively large (*N* = 402 patients in total, *N* = 391 after propensity score matching) and we used propensity score matching to minimize selection bias, we cannot be totally sure the results would be the same, if the full cohort had been analysed; therefore, we aim to conduct a new analysis when data on the full cohort will be available. Of course, the facts 40% is a quite large proportion of the total portion of the full cohort and that results of the propensity matched analyses were not largely different from unadjusted comparison (not report in this article for the sake of simplicity), are encouraging signals the outputs of the analysis are reliable.

Therefore, based on real-world clinical experience of Hospital S. Chiara of Trento, the use of RM for the follow-up of patients carrying ICD and CRT-D appears associated with a reduced mortality for all causes and hospitalizations for cardiovascular causes, thus confirming the most positive findings reported in literature, which is not homogeneous with regard to the benefits associated with RM (20, 21, 24, 30–33).

The reduction of the hospitalization burden generates savings for both the hospital and the regional healthcare system, with respect of a modest increase in costs for the management of remote control. Although these initial observations need further confirmation (broader sample, longer follow-up) the organizational model seems efficient, and the current evidence suggests the strategy can be fine-tuned to become more cost-effective.

## Data Availability

The raw data supporting the conclusions of this article will be made available by the authors, without undue reservation.
