# Supplementary material for: Economic analysis of remote monitoring in patients with implantable cardioverter defibrillators or cardiac resynchronization therapy defibrillators in the Trento area, Italy
Source: Front Cardiovasc Med. 2023 May 25;10:1151167. doi: 10.3389/fcvm.2023.1151167 (PMC10247992; doi:10.3389/fcvm.2023.1151167)

Supplementary Material

**Economic analysis of remote monitoring in patients with Implantable Cardioverter Defibrillators or cardiac resynchronization therapy defibrillators in the Trento area, Italy**

**Massimiliano Marini MD, FHRS, FESC^1*^, Lodovica Videsott MD^1^, Chiara Francesca Dalle Fratte MD^2^, Andrea Francesconi^3^, Eleonora Bonvicini MD^1^, Silvia Quintarelli MD^1^, Marta Martin MD^1^, Fabrizio Guarracini MD, FHRS^1^, Alessio Coser MD^1^, Roberto Bonmassari MD^1^, Giuseppe Boriani MD, FEHRA, FESC^4^.**

^1^Department of Cardiology, S. Chiara Hospital, Trento, Italy

^2^Controlling Department of the APSS, Trento, Italy

^3^Department of Management and Economy, University of Trento, Italy

^4^Cardiology Division, Department of Biomedical, Metabolic and Neural Sciences, University of Modena and Reggio Emilia, Policlinico di Modena, Modena, Italy

*** Correspondence:**Marini Massimiliano
[massimiliano.marini@apss.tn.it](mailto:massimiliano.marini@apss.tn.it)

Table S1. Patients’ charactristics at baseline (unadjusted data)

| **Parameter** | **SM group (N=189)** | **RM group**  **(N=213)** | **Total (N=402)** | **Difference (test)*** |
| --- | --- | --- | --- | --- |
| Age, years, mean (sd) | 69.94 (10.76) | 64.73 (13.20) | 67.18 (12.38) | **p<0.0001** |
| Male gender, % | 77.25% (146/189) | 76.53% (163/213) | 76.87% (309/402) | p=0.864 |
| Diabetes, % | 29.28% (53/181) | 21.36% (44/206) | 25.06% (97/387) | p=0.073 |
| Pulmonary arterial hypertension, % | 65.19% (118/181) | 57.77% (119/206) | 61.24% (237/387) | p=0.135 |
| Severe chronic kidney disease, % | 10.50% (19/181) | 3.38% (7/207) | 6.70% (26/388) | **p=0.005** |
| Secondary cardiovascular prevention, % | 34.39% (65/189) | 35.85 (76/212) | 35.16% (141/401) | p= 0.760 |
| Stroke or TIA, % | 9.94% (18/181) | 7.25 (15/207) | 8.51% (33/388) | p=0.342 |
| Myocardial infarction, % | 42.70% (79/185) | 33.81% (71/210) | 37.97% (150/396) | p=0.069 |
| Coronary disease, without MI, % | 18.13% (33/182) | 13.33% (28/210) | 15.56% (61/392) | p=0.191 |
| Thromboembolism or vasculopathy, % | 9.39% (17/181) | 5.77% (12/208) | 7.46% (29/389) | p=0.175 |
| Deep vein thrombosis, % | 76.13% (118/155) | 70.05% (138/197) | 72.73% (256/352) | p=0.204 |
| Aortic stenosis, % | 5.26% (8/152) | 2.04% (4/196) | 3.45% (12/348) | p=0.102 |
| Atrial fibrillation, % | 36.61% (67/183) | 27.14% (57/210) | 31.55% (124/393) | **p=0.044** |
| Treatment with ACE inhibitors / ARBs, % | 75.00% (129/172) | 77.94% (159/204) | 76.60% (288/376) | p=0.502 |
| Treatment with beta-blockers, % | 89.53% (154/172) | 92.61% (188/203) | 91.20% (342/375) | p=0.295 |
| Treatment with diuretics, % | 86.05% (148/172) | 75.49% (154/204) | 80.32% (302/376) | **p=0.010** |
| Treatment with anticoagulation drugs, % | 35.39% (63/178) | 36.71% (76/207) | 36.10% (139/385) | p=0.788 |
| Treatment with antiarrhythmic drugs, % | 20.48% (34/166) | 19.70% (40/203) | 20.05% (74/369) | p=0.853 |
| Treatment with ICD, % | 52.38% (99/189) | 63.38% (135/213) | 58.21% (234/402) | **p=0.026** |
| Treatment with CRT-D, % | 47.62% (907189) | 36.62% (78/213) | 41.79% (168/402) |  |
| CHA_2_DS_2_-VASc score, mean (sd) | 3.30 (1.57) | 2.86 (1.67) | 3.06 (1.64) | **p=0.0083** |
| Left ventricular ejection score, %  <35%  35-44%  45-54%  >55%; | 72.62% (122/168) 9.52% (16/168)  9.52% (16/168)  8.33% (14/168) | 63.37% (128/202)  11.88% (24/202)  8.42% (17/202) 16.34% (33/202) | 67.57% (250/370) 10.81% (40/370)  8.92% (33/370) 12.70% (47/370) | p=0.094 |
| NYHA Functional Classification, %  Class I  Class II  Class III  Class IV | 16.58% (31/187) 60.43% (113/187) 22.99% (43/187) 0.00% (0/187) | 23.94% (51/213) 65.73% (140/213) 10.33% (22/213) 0.00% (0/213) | 20.50% (82/400) 63.25% (253/400) 16.25% (65/400) 0.00% (0/400) | **p=0.002** |

*t-test for age and CHADS score; chi-square test for all other variables. Statistically significant differences between groups are marked in bold.

ACE: angiotensin-converting enzyme; ARB: angiotensin II receptor blocker; CHA_2_DS_2_-VASc score: Congestive heart failure, Hypertension, Age over 75 years, Diabetes mellitus, Stroke, Vascular disease, Age between 65-74 years, Sex Category (female); CRT-D: cardiac resynchronization therapy defibrillators; ICD: implantable cardioverter-defibrillators; NYHA: New York Heart Association; RM: remote monitoring; SM: standard monitoring.

Figure 4 (S1). Propensity score matching model and matching outcomes


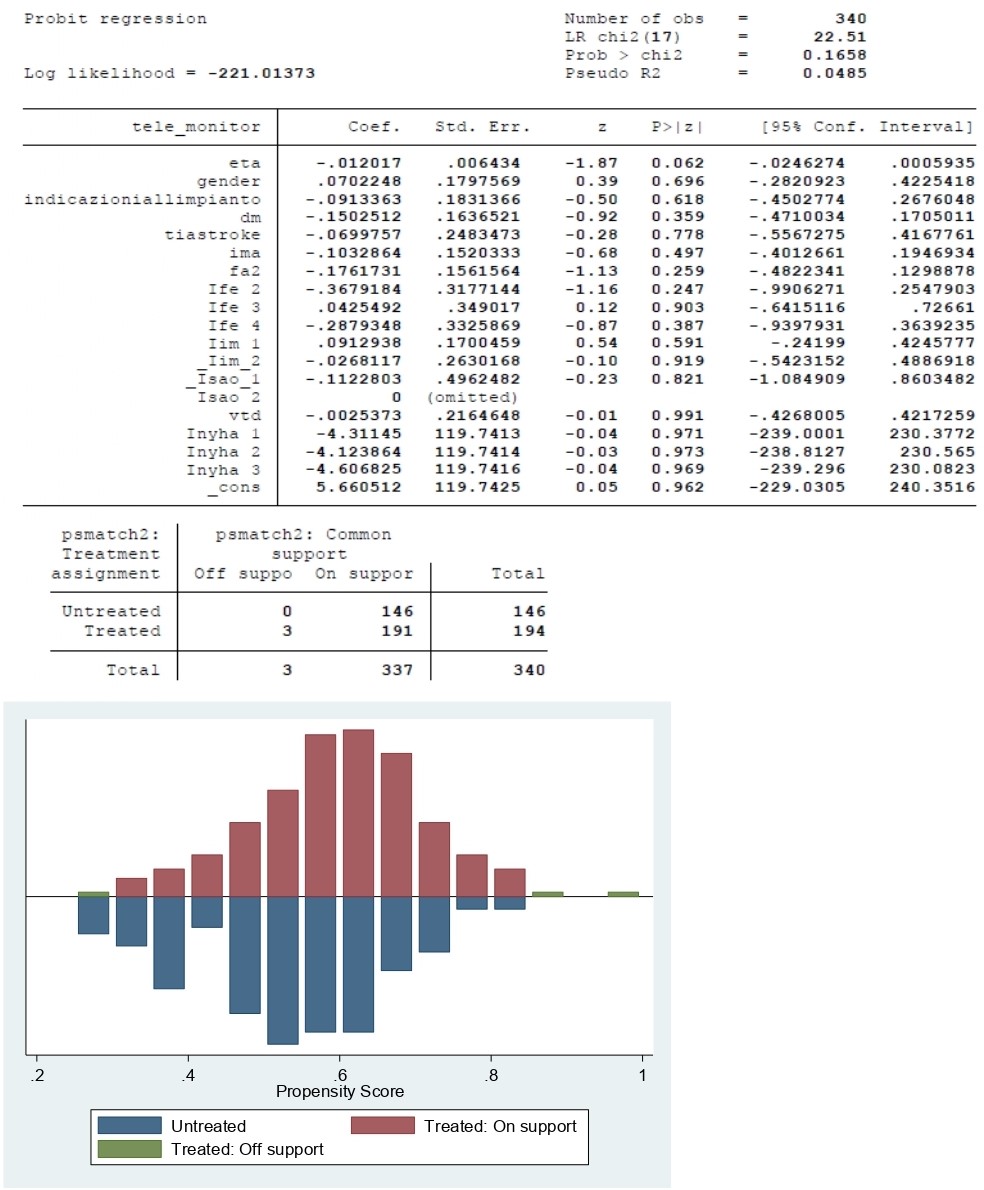

Supplement: Supplementary file 1 [file Datasheet1.docx]
